# Immune dysfunction following COVID-19, especially in severe patients

**DOI:** 10.1038/s41598-020-72718-9

**Published:** 2020-09-28

**Authors:** Cong-Ying Song, Jia Xu, Jian-Qin He, Yuan-Qiang Lu

**Affiliations:** 1 Department of Emergency Medicine and Zhejiang Provincial Key Laboratory for Diagnosis and Treatment of Aging and Physic-Chemical Injury Diseases, The First Affiliated Hospital, School of Medicine, Zhejiang University, 79 Qingchun Road, Hangzhou, 310003 Zhejiang People’s Republic of China; 2 State Key Laboratory for Diagnosis and Treatment of Infectious Diseases, The First Affiliated Hospital, School of Medicine, Zhejiang University, 79 Qingchun Road, Hangzhou, 310003 Zhejiang People’s Republic of China

**Keywords:** Immunology, Microbiology, Diseases, Medical research, Risk factors

## Abstract

The coronavirus disease 2019 (COVID-19) has been spreading worldwide. Severe cases quickly progressed with unfavorable outcomes. We aim to investigate the clinical features of COVID-19 and identify the risk factors associated with its progression. Data of confirmed SARS-CoV-2-infected patients and healthy participants were collected. Thirty-seven healthy people and 79 confirmed patients, which include 48 severe patients and 31 mild patients, were recruited. COVID-19 patients presented with dysregulated immune response (decreased T, B, and NK cells and increased inflammatory cytokines). Also, they were found to have increased levels of white blood cell, neutrophil count, and D-dimer in severe cases. Moreover, lymphocyte, CD4^+^ T cell, CD8^+^ T cell, NK cell, and B cell counts were lower in the severe group. Multivariate logistic regression analysis showed that CD4^+^ cell count, neutrophil-to-lymphocyte ratio (NLR) and D-dimer were risk factors for severe cases. Both CT score and clinical pulmonary infection score (CPIS) were associated with disease severity. The receiver operating characteristic (ROC) curve analysis has shown that all these parameters and scores had quite a high predictive value. Immune dysfunction plays critical roles in disease progression. Early and constant surveillance of complete blood cell count, T lymphocyte subsets, coagulation function, CT scan and CPIS was recommended for early screening of severe cases.

## Introduction

The coronavirus disease 2019 (COVID-19), caused by severe acute respiratory syndrome coronavirus 2 (SARS-CoV-2) infection, has been spread worldwide^1,2^. Because it brought so much damage and negative effects, the World Health Organization (WHO) declared the outbreak a public health emergency of international concern on January 31, 2020^3^. This disease has progressed rapidly, and patients who are in the severe stage could develop acute respiratory distress syndrome, sepsis, and even multiple organ dysfunction syndrome in just a short time^4^. Severe cases had unfavorable outcomes according to the latest epidemiological statistics^5^, which means that early identification and intervention for severe patients were very important, especially because no effective treatment has been made yet directly targeting at SARS-CoV-2. So, we collected and compared data of healthy people and laboratory-confirmed SARS-CoV-2 infected patients. The aim of this study was to know the clinical characteristics of COVID-19 and then identify the independent risk factors related to disease severity and so help clinicians distinguish severe cases by using clinical data in the early stage.

## Results

### Demographic and clinical characteristics of COVID-19 patients

**Figure 1:**
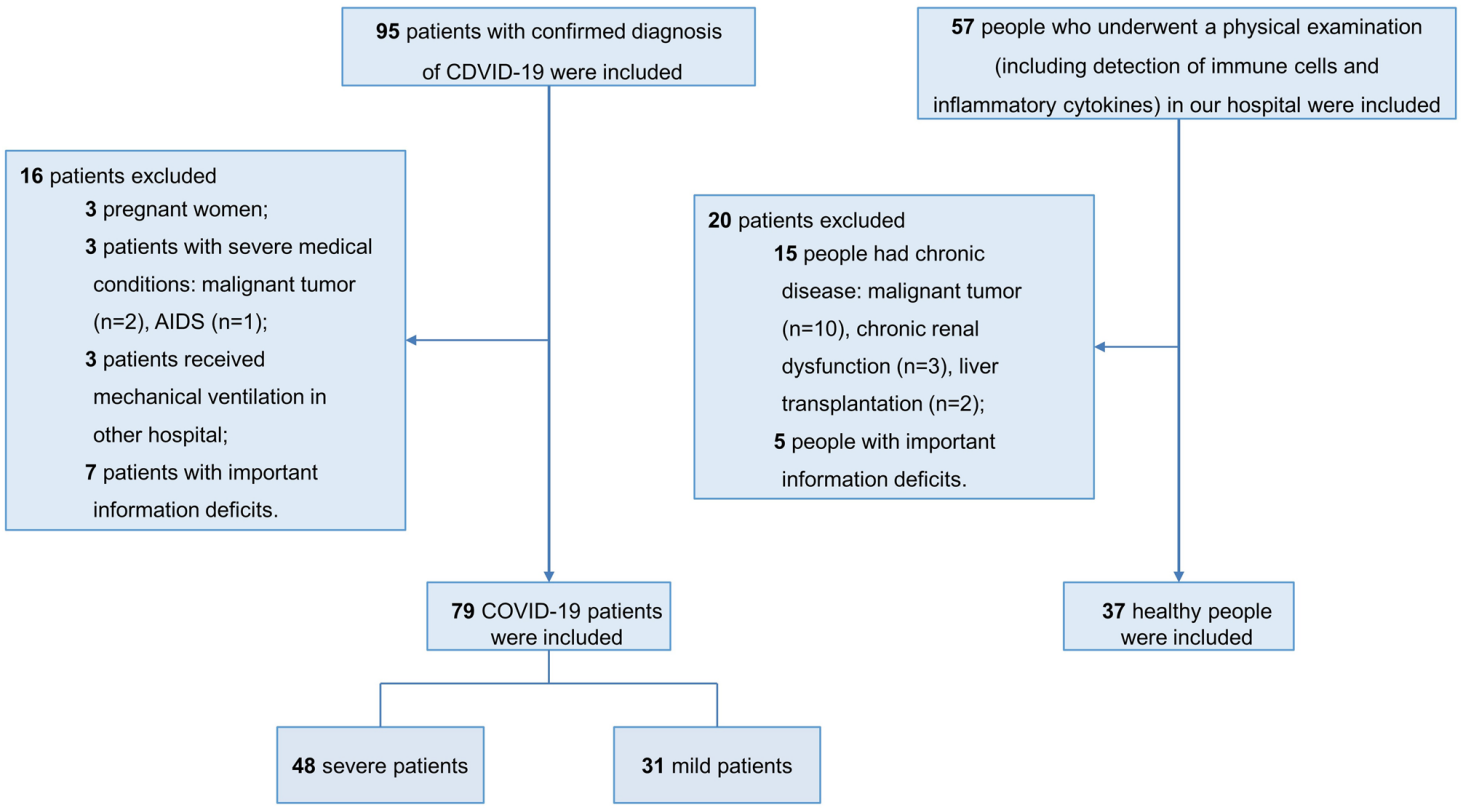
Flow chart of patients screening. Shown were the concrete procedures of patients screening. The whole screening process strictly followed the inclusion criteria and exclusion criteria.

Ninety-five confirmed patients were firstly included, among which 3 pregnant women, 2 patients with malignant tumor, 1 patient with AIDS, 3 patients who have received mechanical ventilation in another hospital, and 7 patients with important information deficits were excluded. Finally, a total of 79 patients were included in the study: 48 severe patients and 31 mild patients. Thirty-seven healthy people who underwent a physical examination (including detection of immune cells and inflammatory cytokines) were included as the healthy control (shown in Fig. 1). As in comparison to healthy people (Table 1), patients with COVID-19 had decreased lymphocyte (0.8 [IQR, 0.5–1.2] vs. 1.9 [IQR,1.3–2.2]; *P* < 0.001) and increased neutrophil-to-lymphocyte ratio (NLR) (5.5 [IQR, 2.3–14.3] vs. 1.9 [IQR, 1.2–2.9]; *P* < 0.001) and C-reactive protein (CRP) (24.3 [IQR, 9.4–51.2] vs. 5.7 [IQR, 1.1–7.9]; *P* < 0.001). SARS-CoV-2-infected patients have problems with liver and kidney function, showing elevated levels of alanine aminotransferase (ALT), aspartate aminotransferase (AST), and creatinine (Cr). Comparing data between mild and severe patients (Table 2), more than half (62.0%) of all confirmed patients were male, and the proportion of males in severe group was higher than that in the mild group; however, there was no statistically significant difference (68.8% vs. 51.6%, *P* = 0.125). Patients in the severe group had a higher age distribution than in the non-severe group (57.0 years [IQR, 51.0–66.0] vs. 48.0 years [IQR, 40.0–57.0]; *P* = 0.006). There was no significant difference in smoking status between severe group and mild group. 93.7% of the COVID-19 patients have shown fever symptoms, and more than half of them (59.5%) had cough. Both expectoration and myalgia or fatigue were also common among them. More severe patients have shown dyspnea (39.6% vs. 3.2%, *P* < 0.001), and severe patients have shown a lower SpO_2_ (96.0% [IQR, 95.0–98.0] vs. 98.0% [97.0–99.0], *P* < 0.001). The percentage of patients with hypertension was higher in the severe group (24 [50.0%] vs. 5 [16.1%], *P* = 0.002), while other coexisting disorders, including diabetes, cardiovascular disease, and chronic obstructive pulmonary disease, have shown no statistically significant differences. In addition, many laboratory items have significant differences. The levels of white blood cell count, neutrophil count, NLR, erythrocyte sedimentation rate (ESR), CRP, and D-dimer were higher in severe patients than in mild patients, while the level of lymphocyte was lower in severe patients. Organ injury indicators such as serum urea nitrogen (UN), lactose dehydrogenase (LDH), and hydroxybutyrate dehydrogenase (HBDH) were also higher in severe patients.

**Table 1:** Comparisons of characteristics between healthy people and COVID-19 patients.

Demographic and clinical characteristics	Healthy people (n = 37)	COVID-19 patients (n = 79)	*P* value
Age (years)	51.0 (38.0–63.5)	54.0 (45.0–63.0)	0.376
**Sex**			
Male	19 (51.4)	49 (62.0)	0.125
Female	18 (48.6)	30 (38.0)	
**Laboratory results**			
White blood cell count (× 10^9^/L)	5.8 (4.8–7.0)	5.7 (3.9–8.7)	0.488
Neutrophil count (× 10^9^/L)	3.4 (2.7–4.5)	4.1 (2.5–7.2)	0.143
Lymphocyte count (× 10^9^/L)	1.9 (1.3–2.2)	0.8 (0.5–1.2)	< 0.001
NLR^a^	1.9 (1.2–2.9)	5.5 (2.3–14.3)	< 0.001
Monocyte count (× 10^9^/L)	0.4 (0.3–0.5)	0.3 (0.2–0.5)	0.087
Platelet count (× 10^9^/L)	214.0 (184.5–251.5)	191.0 (145.0–234.0)	0.013
Haemoglobin (g/L)	134.0 (119.0–147.5)	141.0 (127.0–150.0)	0.182
Red blood cell distribution width (%)	12.9 (12.0–13.6)	12.2 (11.9–12.7)	0.315
C-reactive protein (mg/L)	5.7 (1.1–7.9)	24.3 (9.4–51.2)	< 0.001
Alanine aminotransferase (U/L)	17.0 (11.0–24.3)	22.0 (16.0–37.0)	0.010
Aspartate aminotransferase (U/L)	18.0 (15.5–22.7)	21.0 (17.0–34.0)	0.018
Creatinine (μmol/L)	62.0 (55.5–78.0)	77.0 (63.0–89.0)	0.001
Serum urea nitrogen (mmol/L)	4.5 (3.7–5.5)	5.0 (4.0–7.2)	0.073

Data are presented as medians (interquartile ranges, IQR) or N (%).

^a^*NLR* neutrophil-to-lymphocyte ratio.

**Table 2:** Comparisons of characteristics between severe patients and mild patients.

Demographic and clinical characteristics	Severe patients (n = 48)	Mild patients (n = 31)	*P* value
Age (years)	57.0 (51.0–66.0)	48.0 (40.0–57.0)	0.006
**Sex**			
Male	33 (68.8)	16 (51.6)	0.125
Female	15 (31.3)	15 (48.4)	
**Smoking history**			
Never smoker	39 (81.3)	24 (77.4)	0.620
Former smoker	3 (6.3)	1 (3.2)	
Current smoker	6 (12.5)	6 (19.4)	
**Symptoms**			
Fever	47 (97.9)	27 (87.1)	0.054
Cough	29 (60.4)	18 (58.1)	0.835
Expectoration	16 (33.3)	7 (22.6)	0.304
Headache	6 (12.5)	2 (6.5)	0.384
Myalgia or fatigue	16 (33.3)	7 (22.6)	0.304
Chill	5 (10.4)	1 (3.2)	0.239
Pharyngalgia	2 (4.2)	2 (6.5)	0.651
Dyspnea	19 (39.6)	1 (3.2)	< 0.001
Nausea or vomiting	2 (4.2)	1 (3.2)	0.831
**Exposure history**			
Epidemic area-related exposure	24 (50.0)	16 (51.6)	0.889
Close contact to confirmed patients	19 (39.6)	14 (45.2)	0.624
**Basic vital signs**			
T_max_ (℃)	38.3 (37.5–39.0)	38.0 (37.4–38.7)	0.254
Temperature at hospital admission (℃)	37.0 (36.4–37.7)	37.2 (36.7–37.5)	0.273
Oxygen saturation (%)	96.0 (95.0–98.0)	98.0 (97.0–99.0)	< 0.001
Respiratory (rate breaths/min)	18.5 (18.0–20.0)	18.0 (18.0–20.0)	0.113
Heart rate (beats/min)	84.5 (79.3–95.0)	87.0 (78.0–96.0)	0.928
Mean arterial pressure (mmHg)	100.0 (88.5–108.2)	98.3 (81.7–108.3)	0.343
Days from illness onset to first hospital admission (day)	6.0 (2.0–7.0)	4.0 (2.0–7.0)	0.334
Days from illness onset to initial diagnosis (day)	7.0 (4.0–10.0)	4.0 (3.0–8.0)	0.072
**Coexisting disorders**			
Hypertension	24 (50.0)	5 (16.1)	0.002
Diabetes	9 (18.8)	3 (9.7)	0.273
Cardiovascular disease	5 (10.4)	0 (0)	0.063
Chronic obstructive pulmonary disease	3 (6.3)	1 (3.2)	0.549
Chronic liver disease	6 (12.5)	3 (9.7)	0.700
Length of hospital stay (day)	19.5 (15.0–27.3)	15.0 (12.0–19.0)	0.015
**Laboratory results**			
White blood cell count (× 10^9^/L)	7.0 (4.3–10.7)	4.5 (3.5–6.0)	0.004
Neutrophil count (× 10^9^/L)	6.0 (3.0–9.7)	2.8 (2.0–4.1)	< 0.001
Lymphocyte count (× 10^9^/L)	0.6 (0.4–0.9)	1.1 (0.8–1.3)	< 0.001
NLR^a^	8.2 (3.9–19.2)	3.0 (1.9–5.5)	< 0.001
Monocyte count (× 10^9^/L)	0.3 (0.1–0.5)	0.4 (0.3–0.5)	0.121
Platelet count (× 10^9^/L)	192.0 (163.5–232.3)	187.0 (136.0–239.0)	0.644
Haemoglobin (g/L)	141.5 (128.3–149.8)	141.0 (124.0–150.0)	0.857
Red blood cell distribution width (%)	12.3 (11.9–12.7)	12.0 (11.8–12.7)	0.334
Platelet distribution width (%)	12.4 (11.1–14.4)	12.2 (10.4–13.8)	0.403
Erythrocyte sedimentation rate (mm/h)	48.5 (25.5–66.5)	16.0 (10.0–36.0)	0.001
C-reactive protein (mg/L)	36.9 (16.1–56.7)	11.6 (3.3–27.9)	0.001
Procalcitonin (ng/ml)	0.06 (0.04–0.10)	0.06 (0.03–0.08)	0.400
D-dimer (ug/L)	452.5 (312.0–799.8)	236.0 (170–413.0)	< 0.001
Alanine aminotransferase (U/L)	21.5 (15.0–32.3)	23.0 (16.0–36.0)	0.835
Aspartate aminotransferase (U/L)	21.5 (16.3–41.0)	20.0 (18.0–30.0)	0.619
Creatinine (μmol/L)	79.0 (66.0–90.8)	74.0 (60.0–88.0)	0.164
Serum urea nitrogen (mmol/L)	5.4 (4.6–8.4)	4.3 (3.4–5.2)	< 0.001
Lactose dehydrogenase (U/L)	288.6 (227.3–358.8)	223.0 (183.0–263.0)	0.001
Hydroxybutyrate dehydrogenase (U/L)	234.0 (196.0–292.8)	175.0 (151.0–219.0)	< 0.001

Data are presented as medians (interquartile ranges, IQR) or N (%).

^a^*NLR* neutrophil-to-lymphocyte ratio.

### Treatment and clinical outcomes

Treatment for COVID-19 followed standard therapeutic guidelines. All patients received single or combined antiviral treatment, including lopinavir-ritonavir, arbidol, interferon-α, favipiravir, and darunavir/cobicistat. Seventeen (54.8%) mild patients had short-term application of low-dose glucocorticoids, and 45 (93.8%) severe patients received glucocorticoids. Twenty-two (45.8%) severe patients received antibiotic treatment. All patients received nasal oxygen, among which, 3 severe patients underwent endotracheal intubation, and three critically ill patients underwent intubation combined with ECMO. After systematic isolation treatment, all patients got negative results of rRT-PCR for SARS-CoV-2 and discharged. Severe patients had a longer hospital stay than mild patients (19.5 [IQR, 15.0–27.3] vs. 15.0 [12.0–19.0], *P* = 0.015). No deaths had occurred during hospital stay.

### Immune status of patients with COVID-19

**Figure 2:**
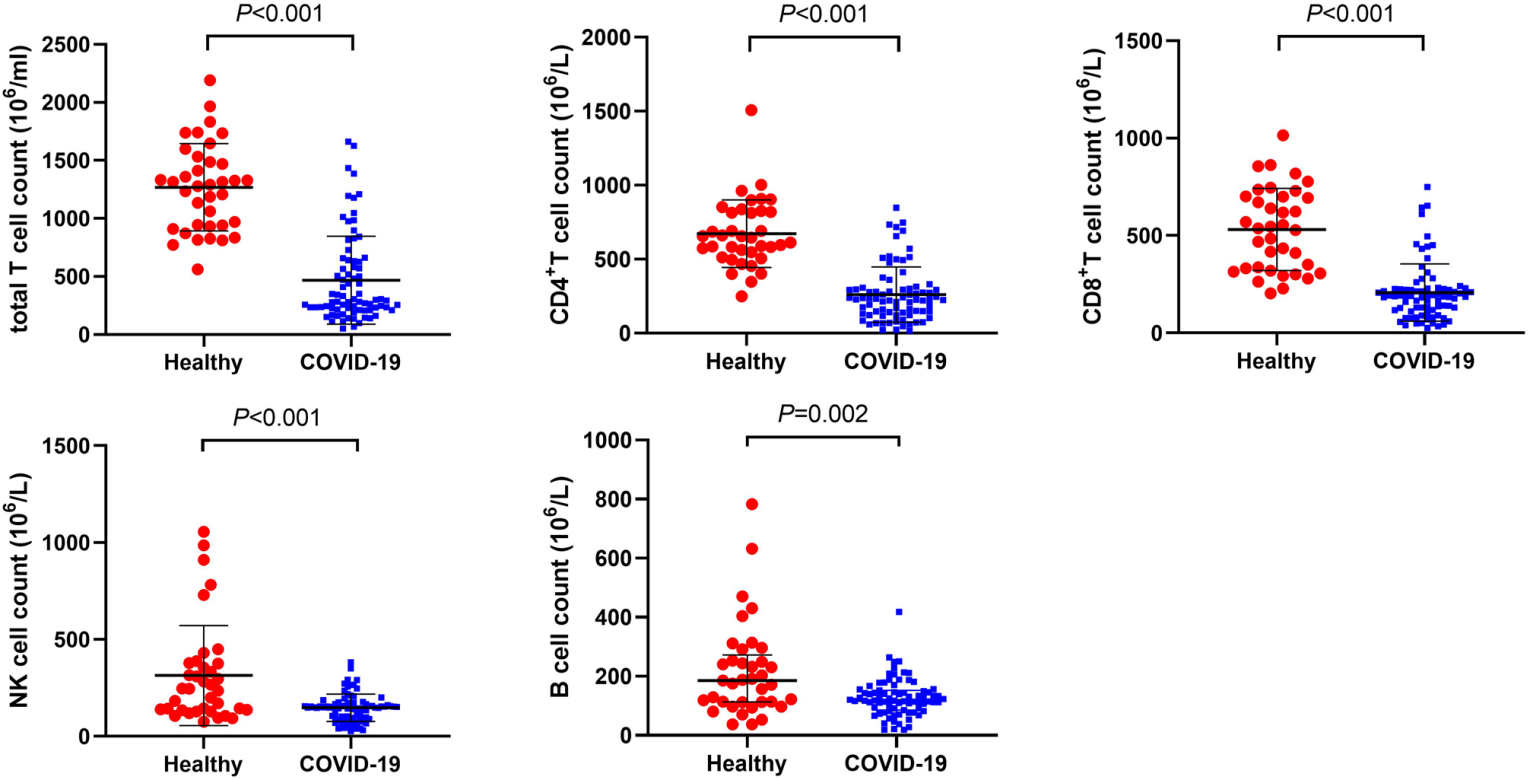
Immune cell counts in healthy people and COVID-19 patients. Immune cell counts, including total T cell, CD4^+^ T cell, CD8^+^ T cell, NK cell, and B cell were compared between healthy people and COVID-19 patients. All these immune cell counts were lower in COVID-19 patients.

**Figure 3:**
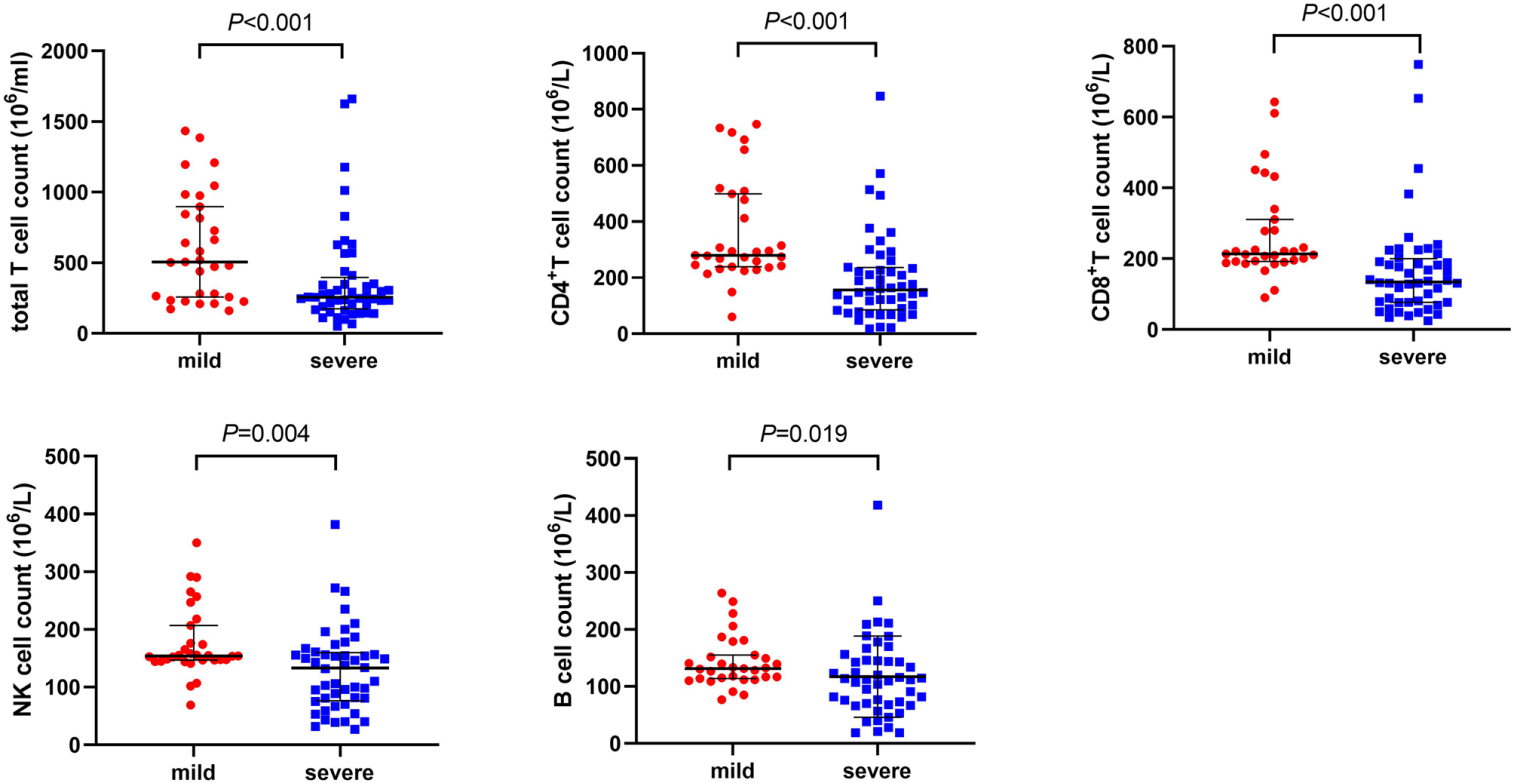
Immune cells count in mild and severe COVID-19 patients. Immune cell counts, including total T cell, CD4^+^ T cell, CD8^+^ T cell, NK cell, and B cell were compared between mild and severe COVID-19 patients. All these immune cell counts were lower in severe COVID-19 patients.

**Figure 4:**
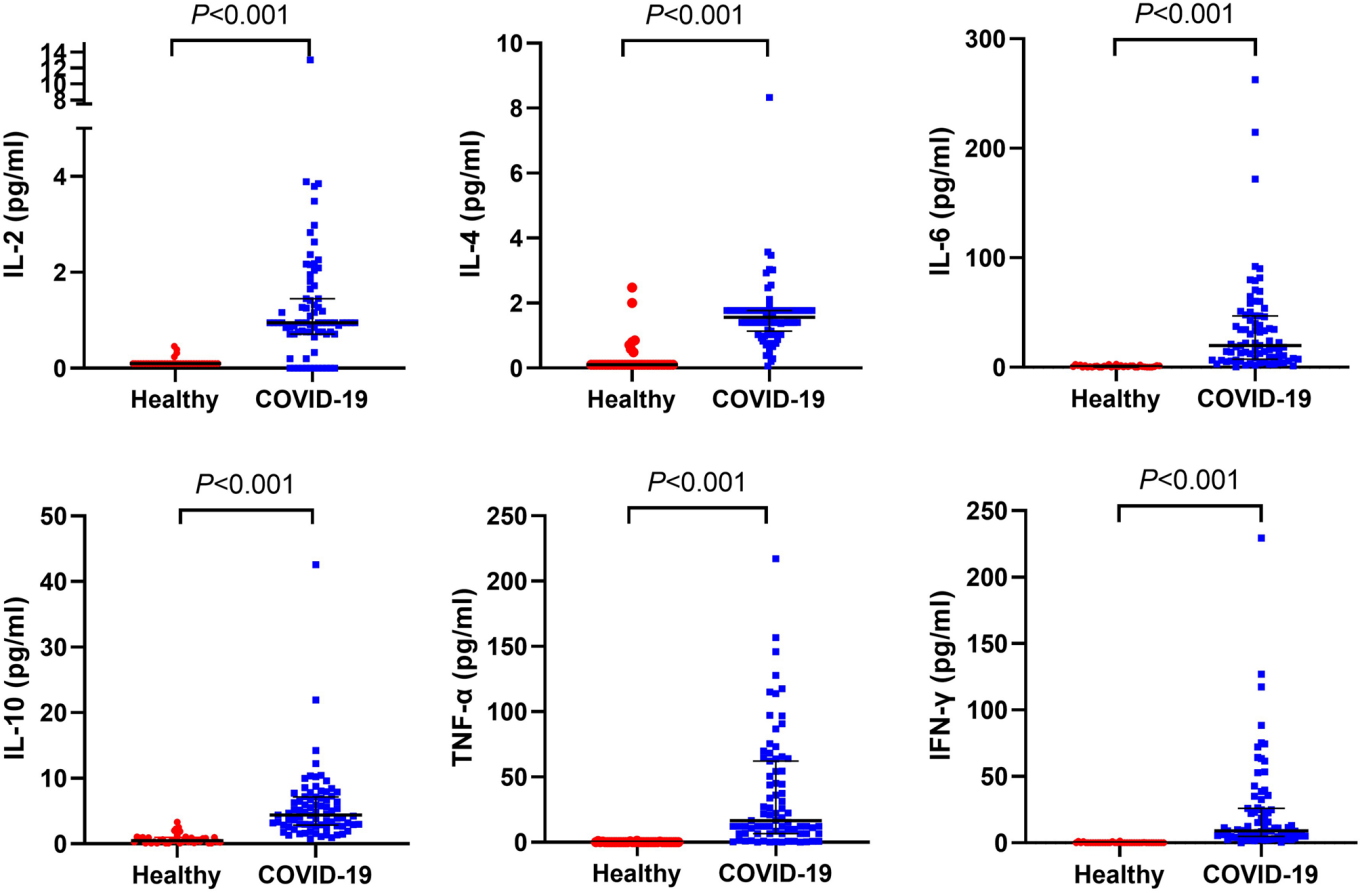
Inflammatory cytokines in healthy people and COVID-19 patients. Inflammatory cytokines, including IL-2, IL-4, IL-6, IL-10, TNF-α, IFN-γ were compared between healthy people and COVID-19 patients. All these inflammatory cytokines were higher in COVID-19 patients.

**Figure 5:**
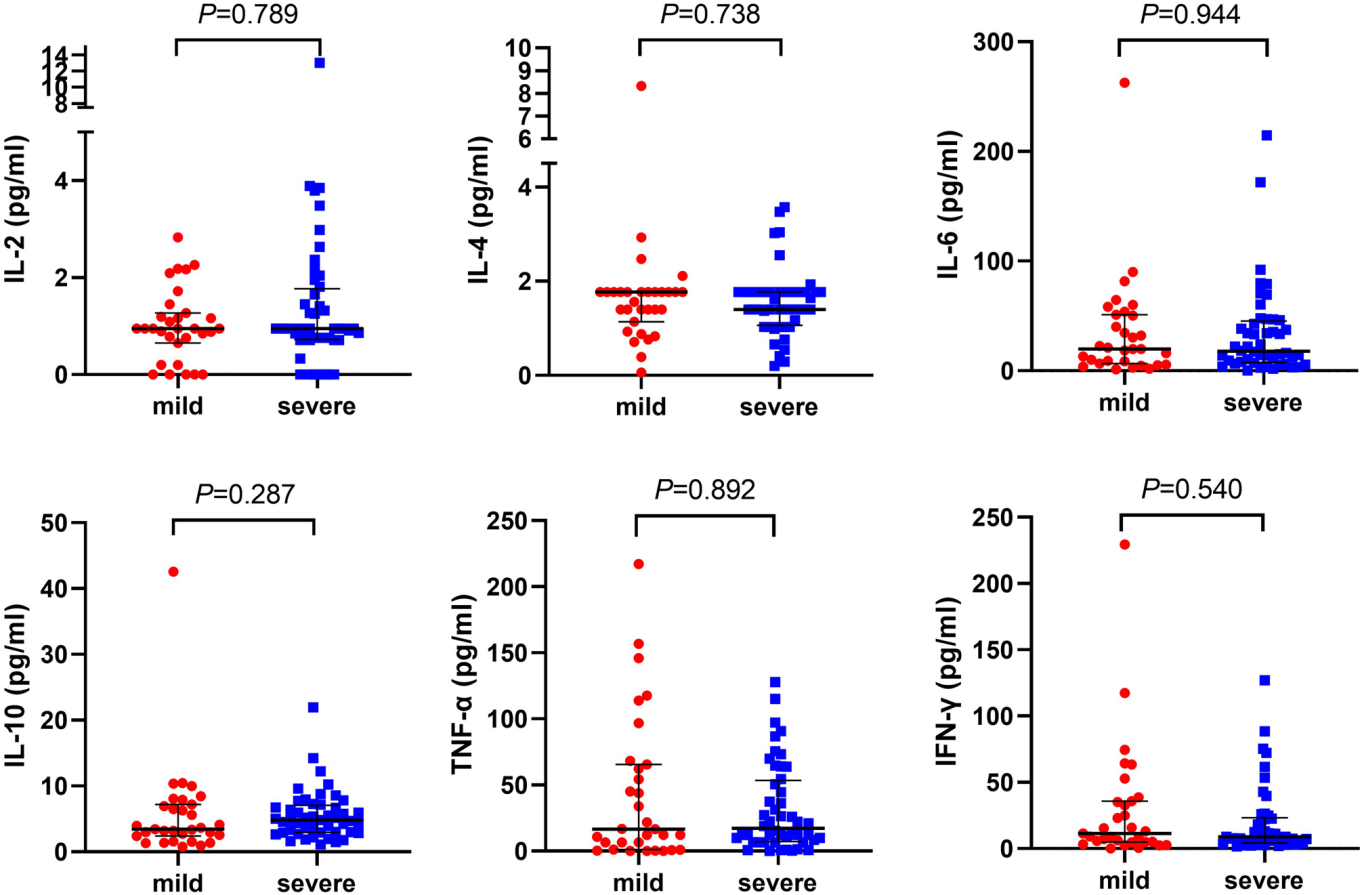
Inflammatory cytokines in mild and severe COVID-19 patients. Inflammatory cytokines, including IL-2, IL-4, IL-6, IL-10, TNF-α, IFN-γ were compared between mild and severe COVID-19 patients. There was no significant difference between two groups.

Immune cell count and inflammatory factors were recorded to know the immune status of COVID-19 patients. They had a decreased immune cell count, including total T cell, CD4^+^ T cell, CD8^+^ T cell, and NK cell and B cell count as in comparison with healthy people (Fig. 2), and the levels of these immune cell count were also lower in severe than in mild cases (Fig. 3). There are higher levels of inflammatory factors, including IL-2, IL-4, IL-6, IL-10, TNF-α, and IFN-γ, found in COVID-19 patients than in healthy people (Fig. 4). However, these levels have no significant difference between severe and mild cases (Fig. 5). We also compared immune status between intubation group (n = 6) and non-intubation group. Intubated patients had lower CD4^+^ cell and CD8^+^ cell counts than patients without intubation. While, inflammatory factors had no significant differences between two groups.

### Independent risk factors for severe COVID-19 cases

After initial analysis, variables with *P* < 0.05 were selected, and multivariate logistic regression analysis was performed with the use of the forward stepwise method. Afterward, the CD4^+^ cell count (*P* = 0.015), NLR (*P* = 0.032) and D-dimer (*P* = 0.016) were considered the independent risk factors of the severe COVID-19 cases (Table 3). Then, the following equation was derived: Probability (severe COVID-19) = 1/1 + exp − [− 0.483 + (− 0.005 × CD4^+^ T cell count) + (0.111 × NLR) + (0.003 × D-dimer)]. As for intubated patients and non-intubated patients, increased D-dimer was the only independent risk factor for intubation.

**Table 3:** Multivariable associations between the predictor variables and COVID-19 severity.

Parameters	Beta	χ^2^ value	OR (95% CI)	*P* value
CD4^+^ T cell count (10^6^/L)	− 0.005	5.940	0.995 (0.991–0.999)	0.015
NLR^a^	0.111	4.599	1.117 (1.010–1.236)	0.032
D-dimer (ug/L)	0.003	5.785	1.003 (1.001–1.006)	0.016
Constant	− 0.483	0.387		

^a^*NLR* neutrophil-to-lymphocyte ratio.

### Predictive value of CT score, CPIS and other clinical parameters

**Figure 6:**
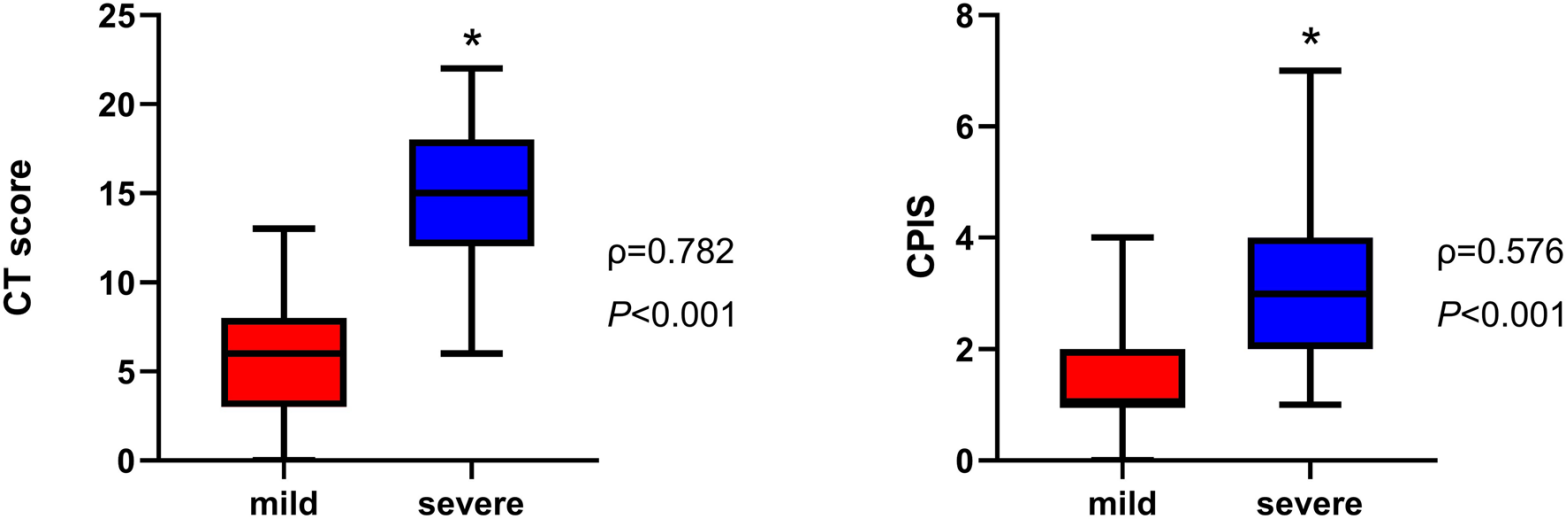
CT score and CPIS in mild and severe COVID-19 patients. CT score and CPIS were calculated and compared between mild and severe COVID-19 patients. CT score and CPIS were higher in severe patients and these two clinical scores were positively related to disease severity.

**Figure 7:**
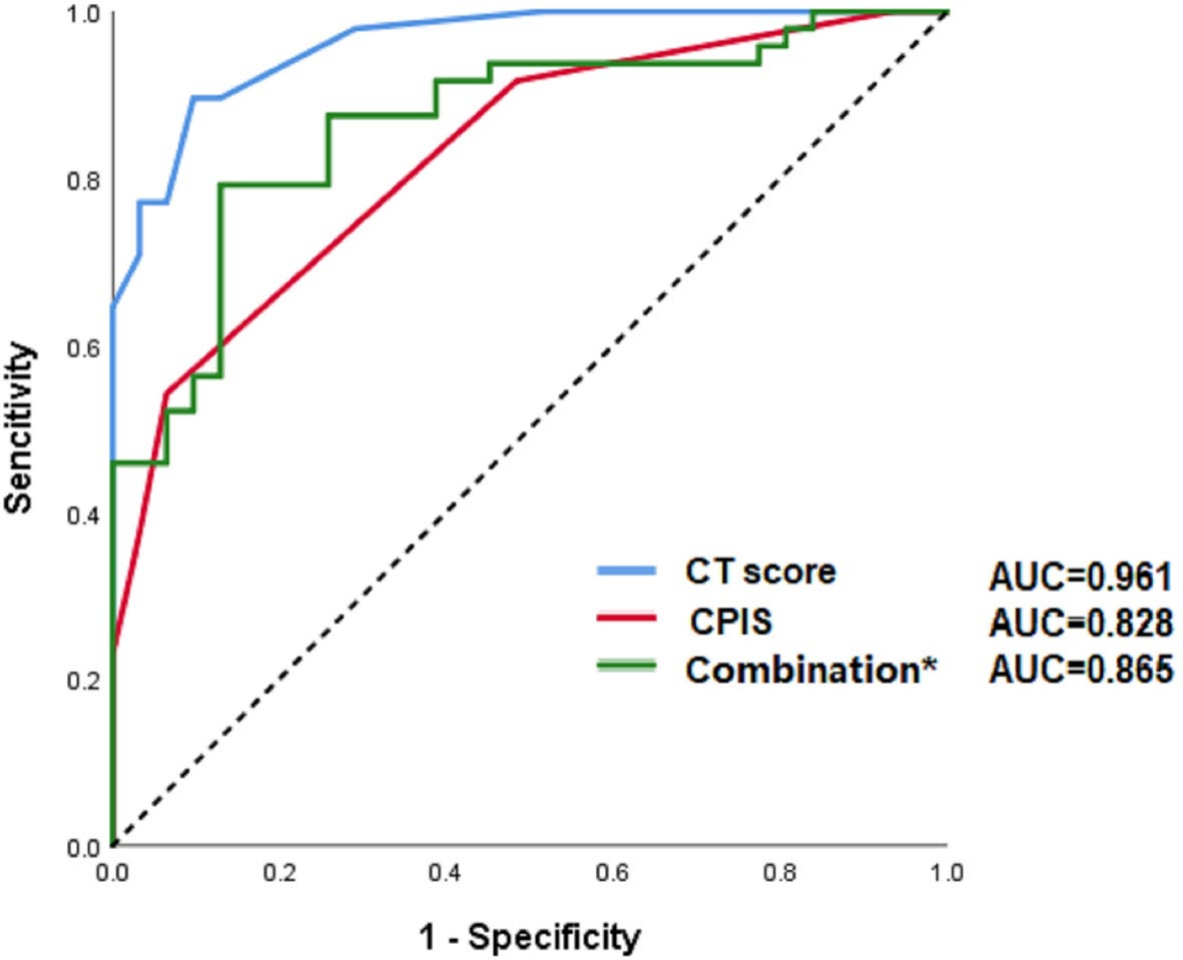
Receiver operating characteristic curves. ROC curve analysis showed CT score, CPIS, and combination of three clinical parameters (CD4^+^ T cell count, D-dimer, and NLR) had good predictive value, in which, CT score had the highest AUC. *Combination: combination of CD4^+^ T cell count, D-dimer, and NLR.

Figure 6 has shown that severe patients had higher CT score and CPIS. According to the results of Spearman’s rank-order correlation analysis, both CT score and CPIS were positively correlated with disease severity (ρ = 0.782, *P* < 0.001; ρ = 0.576, *P* < 0.001, respectively). Intubated patients also had higher CT score (18.0 [16.0–20.0] vs. 10 [6.0–15.0], *P* = 0.002), and higher CPIS than others (4.5 [3.5–5.5] vs. 2.0 [1.0–3.0], *P* = 0.005). To evaluate the predictive value of CT score, CPIS, and three independent risk factors, the ROC curve analysis was performed (Fig. 7). To better distinguish severe and non-severe patients, we have defined the new threshold value of these parameters by calculating the cut-off value. Table 4 has shown that CT score had the greatest predictive value with an AUC of 0.961 (95%CI, 0.925–0.997). CPIS as well as the combination of CD4^+^ T cell count, NLR, and D-dimer had an AUC of 0.828 (95% CI, 0.738–0.917) and 0.865 (95%CI, 0.784–0.946), respectively. The optimal cut-off values of the CT score and CPIS were 9.50 and 2.50, respectively. They all had a quite high sensitivity and specificity at the optimal cut-off value. These results have shown that the said parameters had quite a high predictive value.

**Table 4:** Cut-off values of risk factors associated with disease severity.

Parameters	AUC	Cut-off Value	95% CI	Sensitivity	Specificity
CT score	0.961	9.50	0.925–0.997	0.896	0.903
CPIS	0.828	2.50	0.738–0.917	0.542	0.935
Combination of three parameters	0.865	0.55	0.784–0.946	0.792	0.871

## Discussion

The worldwide outbreak of COVID-19 has worsened^6^. As the main battlefield during the first stage, early detection and effective quarantine of patients and close contacts have allowed the epidemic in China so far to be under effective control. However, the mortality rate of COVID-19 patients in the severe group remains to be quite high because of the rapid progression of the disease and because there is no specific drug against the virus. In-depth research on the characteristics of severe cases was urgently needed to identify severe individuals earlier and quicker. In our study, we compared clinical characteristics between healthy people and COVID-19 patients, and then compared these features between severe and mild cases. We found immune dysfunction in COVID-19 patients, and immunosuppression was more obvious in severe cases than mild cases. Parameters including CD4^+^ T cell count, NLR, and D-dimer, CT score, and CPIS had quite great value for predicting disease severity, which could be considered in early warning of severe patients.

Severe cases usually have mild symptoms in the first week. The time point of aggravation was usually 9 days to 12 days after illness onset, after which the disease progressed quickly^5^. With the characteristics of the disease course, the first week was regarded as the early stage of the disease. So, we collected early clinical data 8 days from illness onset. In this study, we have identified the independent risk factors for severe cases, such as decrease of CD4^+^ T cell count and increase of NLR and D-dimer. NLR was a great indicator of the overall immune status^7^ and was a widely used marker to assess the severity of bacterial infections and the prognosis of patients with pneumonia and tumors^8–10^. Several studies have shown that severe SARS-CoV-2-infected patients have a higher NLR^11,12^, an independent risk factor for mortality in COVID-19 patients^13^. D-dimer was a molecular marker of hypercoagulable state and hyperfibrinolysis, and it could be used in the prognosis of patients with infection or sepsis^14^. In patients with sepsis, inflammatory cells were activated, leading to the activation of the coagulation cascade and then causing the activation of the fibrinolytic system^15^. Increased coagulation could be found in COVID-19 patients, and increased D-dimer was associated with poor prognosis in COVID-19 patients^5,16^. In our study, we also found increased D-dimer was an independent risk factor for intubation. Consistent with a previous study, we found lymphocyte, CD4^+^ T cell, CD8^+^ T cell, NK cell, and B cell counts to be negatively correlated to the severity of COVID-19, suggesting that immune suppression could be more likely found in severe patients and SARS-CoV-2 may directly or indirectly damage the lymphocytes or NK cells and thus further aggravate the disease progression^17,18^. Even though inflammatory cytokine storms were thought to be a mechanism for COVID-19 progression^19–21^, there was only an increase in IL-2, IL-4, IL-6, IL-10, TNF-α, and IFN-γ when comparing pneumonia patients with healthy people, but no significant increase in severe patients compared to mild patients. Thus, the role of inflammatory cytokine storms in the progression of the disease was still unclear and controversial. However, these findings might also be due to the limitation in size and the large heterogeneity at the time points of the first detection. IL-1β is a key proinflammatory cytokine in pyroptosis, which played an important role in various infectious diseases. It was reported that IL-1β increased in COVID-19 patients and was associated with the disease severity^22,23^. However, this item was not detected in our hospital, thus, we could not explore the predictive value of IL-1β in COVID-19 patients. And it could be considered to detect the level of IL-1β in further clinical work or research in order to explore its potential clinical value.

There are similarities of other clinical features of patients in our medical center and those reported in previous studies^24–28^. Males were more susceptible to COVID-19 but had no significance to predict disease severity in our study which may be because of the small sample size, although the male-to-female ratio was quite high in the severe group (68.8 vs. 31.3). Most patients had initial symptoms of fever and cough. The media oxygen saturation was lower in severe group (96.0 [95.0–98.0] vs. 98.0 [97.0–99.0], *P* < 0.001). However, they were all in the normal range, so we did not select this parameter as a predictive factor. It did not mean oxygen saturation had no clinical value. In our study, all data of basic vital signs we selected was the first records on admission, and the first records of oxygen saturation (SpO_2_) was detected by noninvasive pulse oximeter, which was easy to get but could be affected by various interference factors. So it might bring a slight error in this data. Continuous oxygen saturation monitoring or referring to arterial oxygen saturation (SaO_2_) might better reflect the real hypoxemia status of the patient. As for the laboratory results, the higher levels of ESR, CRP, UN, LDH, and HBDH were found in severe COVID-19 patients, suggesting that there is an association between disease progression and the injury of cellular immunity, cardiomyocytes, the liver, and the kidney. Even though these parameters were not independent risk factors based on our analysis, they could be used in severe case screening, and their predicted value should be assessed by using a larger amount of data in further studies.

To investigate whether there was any clinical scoring tool used in early warning for severe cases, we have calculated the CT score and CPIS. Because of its good imaging data reflecting pulmonary inflammation, CT was often used to know whether the COVID-19 case was severe or not. In order to quantify the image data, we have chosen a commonly used CT scoring method to calculate the specific value. CPIS was initially developed as a diagnostic tool for ventilator-associated pneumonia, and it has been used as a predictor of prognosis recently as well^29^. Severe patients had higher CT score and CPIS, and there was a good correlation between these two clinical scores and disease severity. The ROC curve analysis has shown that CT score, CPIS, and combination of clinical parameters had a good predictive value of distinguished severe cases in early stage. Intubated patients also showed higher CT score and CPIS, which suggested these two clinical scoring tools can also be used in pre-intubation evaluation.

There are currently no specific antiviral therapies for SARS-CoV-2 infection. Since immune status play important role in disease severity, immunotherapies are used in severely ill patients^30^. Recent immunotherapies included drugs target specific Inflammatory molecules and pathways, intravenous immunoglobulin therapy, convalescent plasma infusion, and immune cell-targeted therapies (Treg cell and NK cell)^30^. Immunotherapies could regulate abnormal inflammatory response and prevent lung damage. Several researches have suggested immunotherapies can bring clinical benefits, including reduction of viral loads, and improved survival^31,32^. However, the evidence was limited, and the efficacy of these treatments was still not clear, and more researches are needed.

There were several limitations in our study which should be considered. Firstly, it was a retrospective study, which might contain selection bias, but we tried to avoid the bias by abiding strictly to the inclusion and exclusion criteria. Besides that, additional multicenter, multi-ethnic, and prospective studies are expected to revise our diagnostic model, and we also plan to have a multicenter study with a larger sample size so as to further validate and optimize the model. Moreover, it is our hope that better statistical algorithms will make the diagnostic model even more practical. Now, we are trying to develop a COVID-19-related database, making data share and management more efficient. Thus, data from the multicenter study could be used for further analysis.

## Conclusion

The combination of CD4^+^ T cell count, NLR, and D-dimer, CT score, and CPIS could be used as COVID-19 disease severity predictors. We have recommended that these parameters be surveyed earlier and constantly for early warning of severe COVID-19 patients. Immune dysfunction plays a critical role in disease progression. The underlying mechanism of COVID-19 development and progression might be complex, and so further research was urgently needed to help better understand and control this epidemic.

## Methods

### Selection of participants

We included laboratory-confirmed SARS-CoV-2-infected patients in the First Affiliated Hospital, School of Medicine, Zhejiang University between January 20 and February 19, 2020. COVID-19 patients who met any of the following criteria were excluded: (1) pregnant women or patients ages ≤ 18 years; (2) patients who had received mechanical ventilation in other hospitals upon admission; (3) patients having severe medical conditions, including malignant tumor, liver cirrhosis, chronic renal dysfunction, and acquired immune deficiency syndrome (AIDS); and (4) patients with important information deficits. Comparing the characteristics of healthy people and COVID-19 patients, healthy people who underwent a physical examination in our hospital were included as the healthy control group, but people who had a chronic disease or had important information deficits were excluded. Based on the New Coronavirus Pneumonia Prevention and Control Program (7th edition) published by the National Health Commission of China, severity was defined^33^. The mild group referred to patients who have no pneumonia or mild pneumonia. Severe cases, on the other hand, referred to patients with severe pneumonia and who have hypoxia or dyspnea or patients needing ventilatory support and having multiple organ failure.

### Data collection

Demographic and clinical data came from the electronic medical record system of the First Affiliated Hospital, School of Medicine, Zhejiang University. Epidemiological exposure history within the 14 days before the onset of illness is defined as having exposure in the epidemic area and having recently lived, traveled, or had close contact with someone who has been to the epidemic area; (2) close contact is defined as having a contact with a COVID-19-confirmed patient. All laboratory data used in this study were the first in-hospital results right after admission. This study was approved by the Ethical Committee of the First Affiliated Hospital, School of Medicine, Zhejiang University (code number IIT20200025A).

### Laboratory confirmation

Laboratory confirmation was achieved using the real-time reverse transcription-polymerase chain reaction (RT-PCR) assay for SARS-CoV-2 in accordance to the protocol established by the WHO^34^. In our hospital, sputum samples were the sample of choice for RT-PCR assay within 3 h. Two target genes of SARS-CoV-2 were tested during the process: open reading frame 1ab (ORF1ab) and nucleocapsid protein (N).

### CT score and CPIS

The CT scores were analyzed retrospectively by two radiologists without the knowledge of the patient’s diagnosis and other clinical features. The first CT imaging on admission was selected and calculated using the method introduced by Casarini et al.^35^. In order to assess lung infection more rapidly, we have used the simplified version of CPIS^36^, and it was calculated by using the first clinical results in the hospital.

### Statistical analysis

Continuous variables were expressed as medians with interquartile ranges (IQR), while categorical variables were expressed as numbers and percentages in each category. The Mann–Whitney U-test evaluated continuous data, and the chi-square test was used for categorical variables. Performed multivariate logistic regression analyses with forward stepwise method identified independent risk factors. Spearman's rank-order correlation investigated whether the two clinical scores (CT score, CPIS) and disease severity were associated. The predictive powers of these parameters and two clinical scores were known by calculating the area under the receiver operating characteristic curve (AUC). All statistical analyses were done by the SPSS statistical software package (version 25.0). A *P* value < 0.05 means statistically significant.

### Ethics approval and written informed consent

This study was approved by the Ethical Committee of the First Affiliated Hospital, School of Medicine, Zhejiang University (code number IIT20200025A). Written informed consent was obtained from each patient or his/her authorized representatives following a full explanation of the study. All methods and procedures in this study were carried out in accordance with relevant guidelines and regulations.

## Data Availability

The datasets used and analyzed during the current study are available from the corresponding author on reasonable request.
